# Treatment-Related Prognostic Factors in Managing Osteosarcoma around the Knee with Limb Salvage Surgery: A Lesson from a Long-Term Follow-Up Study

**DOI:** 10.1155/2019/3215824

**Published:** 2019-05-02

**Authors:** Jianping Hu, Chunlin Zhang, Kunpeng Zhu, Lei Zhang, Tao Cai, Taicheng Zhan, Xiong Luo

**Affiliations:** Department of Orthopedic Surgery, Shanghai Tenth People's Hospital, Tongji University School of Medicine, 301 Yanchang Middle Road, Shanghai 200072, China

## Abstract

**Purpose:**

The aim of this study was to assess the treatment-related factors associated with local recurrence and overall survival of patients with osteosarcoma treated with limb-salvage surgery.

**Patients and Methods:**

Treatment-related factors were analyzed to evaluate their effects on local recurrence-free survival (LRFS) and overall survival (OS) in 182 patients from 2004 to 2013.

**Results:**

The mean length of follow-up was 73.4 ± 34.7 months (median, 68 months; range, 12-173 months), and 63 patients died by the end of the follow-up. The 5-year and 10-year overall survival rates were 68.6 ± 6.6% and 59.4 ± 10.6%, respectively. Univariate analysis showed that treatment-related prognostic factors for overall survival were prolonged symptom intervals >=60 days, biopsy/tumor resection performed by different centers, previous medical history, incomplete preoperative chemotherapy (<8 weeks), and prolonged postoperative interval >21 days. In the multivariate analysis, biopsy/tumor resection performed by different centers, incomplete implementation of planned new adjuvant chemotherapy, and delayed resumption of postoperative chemotherapy (>21 days) were risk factors for poor prognosis; biopsy/tumor resection performed by different centers and tumor necrosis <90% were independent predictors of local recurrence.

**Conclusion:**

For localized osteosarcoma treated with limb-salvage surgery, it is necessary to optimize timely standard chemotherapy and to resume postoperative chemotherapy to improve survival rates. Biopsies should be performed at experienced institutions in cases of developing local recurrence.

## 1. Introduction

Osteosarcoma is a very rare malignant bone tumor with a high predilection for the area surrounding the knee joint [1]. Since the 1980s, neoadjuvant chemotherapy with limb-salvage surgery (LSS) has become the current standard for the management of high-grade osteosarcomas. Amputation offers better local control, but it confers no clear survival benefit over LSS [2–4], whether in patients with osteosarcoma who respond poorly to chemotherapy or not. The goal of LSS is to preserve a functioning limb without increasing the risk of tumor recurrence. However, there is still concern about the adverse impact of limb-salvage surgery on local recurrence and overall survival due to closer resection margins [5–8].

For those patients with high-grade osteosarcoma around the knee without initial metastases, the surgical margin and the presence of tumor necrosis are generally accepted prognostic factors for local recurrence and overall survival [1, 9–17]. The prognostic relevance of other factors, such as tumor site and size, histologic subtype, neurovascular infiltration, elevated alkaline phosphatase (ALP), elevated lactate dehydrogenase (LDH), and age, is still controversial [18–22]. Other treatment-related factors, such as symptom intervals (SI) [23, 24], incomplete preoperative chemotherapy [25, 26], and delay of resumption of postoperation chemotherapy [27, 28], are still not clear. Because of these contradictory reports, the relevance of treatment-related prognostic factors in patients with osteosarcoma needs further elucidation, especially for those patients who prefer limb sparing. Indeed, the identification of patients with a favorable prognosis and of those with a poor prognosis may be used to adjust treatment and surveillance schedules [29, 30]. This is particularly important for patients who are at risk for local recurrence and inferior survival who have been treated with limb sparing. A reasonable accurate estimate of overall survival and development of local recurrence before treatment decision-making would be helpful to avoid upsetting patients and their parents or confusing them with impractical hope. Therefore, we want to assess the influence of several treatment-related prognostic factors in a large series of patients treated with limb-salvage surgery from East China in long-term follow-up, excluding those patients with primary metastases, which may lead to paradoxical conclusions. We wish to pay more attention to the timing of planned new adjuvant chemotherapy and resumption adjuvant chemotherapy and to try and identify the time intervals, including symptom interval, preoperative chemotherapy duration, and preoperative interval and postoperative interval, which can serve as prognostic factors.

## 2. Patients and Methods

We conducted a retrospective, single-center review of patients with osteosarcoma around the knee after limb-salvage surgery between January 2004 and December 2013. During that time, 254 patients were treated for osteosarcoma around the knee. The study was approved by the Institutional Review Board. The exclusion criteria included metastatic disease at initial presentation (28 patients), limb amputation as the primary procedure (17 patients), initial age >=60 years (14 patients), and incomplete follow-up records (13 patients). 182 patients were included in the final analysis.

### 2.1. Assessment of Patient, Tumor, and Treatment-Related Variables

Age, sex, tumor site, symptom interval, pathological fracture status, previous biopsy history, previous medical history, serum alkaline phosphatase level, preoperative chemotherapy duration, preoperative interval, bone margin length, histologic response (tumor necrosis), tumor size, neurovascular infiltration, postoperative interval, presence of local recurrence, metastatic disease, and overall survival were recorded for each patient (Table 1). For the 182 patients, the median age at diagnosis was 18 years (mean, 21.2 ± 7.6 years; range, 12 to 59 years). There were 114 male patients (62.6%; median age, 18 years; range, 12 to 59 years) and 68 female patients (37.4%; median age, 19 years; range, 12 to 55 years). 103 osteosarcomas (56.6%) were detected in the second, 50 (27.5%) in the third, and 29 (15.9%) in the fourth decade of life or later. The average symptom interval was 68.2 days (median, 65 days; range, 23 to 750 days), and there were 98 patients with a prolonged SI of more than 60 days. Pathologic fracture occurred in 8 patients (4.4%) before primary surgery, 31 patients (17%) underwent biopsy outside of our hospital, and 17 patients (9.3%) presented with previous medical history. Regarding the histological subtypes, there were 134 (73.6%) osteoblastic osteosarcomas, 24 (13%) chondroblastic osteosarcomas, 10 (5.5%) fibroblastic osteosarcomas, and 14 (7.9%) telangiectatic osteosarcomas.

Symptom interval (SI) was defined as the time from the first onset of symptoms or signs to a definitive diagnosis and initiation of treatment [24].

Previous medical history was defined as the patients having accepted inadequate treatment due to an erroneous diagnosis, including erroneous drug and surgical treatment, before they were referred to professional musculoskeletal oncologists. Patients diagnosed with secondary osteosarcoma were also assessed and included in this group.

At diagnosis, the serum ALP levels were measured in international units (IU), and the activity of ALP was estimated by the p-nitrophenyl phosphate method. ALP ranges of 60.0-300.0 IU/L for patients ≤14 years and 38.0-115.5 IU/L for patients >15 years were considered normal.

Responses to neoadjuvant chemotherapy (tumor necrosis) were graded on the basis of the amount of tumor necrosis in the resected specimen. More than 90% tumor necrosis was regarded as a good response; a cut-off of 90% tumor necrosis is usually used to distinguish good and poor responders [31].

Standard chemotherapy followed the protocols of IOR/OS-4 [32]. The standard length of preoperative chemotherapy duration was 10 weeks of 2 cycles of multidrug therapy. The preoperative interval was defined as the time from the end of preoperative chemotherapy to the primary surgery.

Neurovascular infiltration was defined as tumor growth outside of the normal compartment and infiltration of soft tissue, such as the nerve and vasculature, which can be inspected by enhanced MRI preoperatively and confirmed during surgery and by pathological examination. Tumors measuring at least 10 cm in length of the involved bone or more were defined as large and all others as small.

The postoperative interval was defined as the time from primary surgery to the resumption of postoperative chemotherapy.

### 2.2. Diagnosis, Treatment, and Surveillance

Diagnosis of osteosarcoma, established by clinical and radiological findings, was confirmed on core needle biopsy or open biopsy instead of fine biopsy. Thereafter, 2 cycles of neoadjuvant chemotherapy were given to patients. The chemotherapy protocol was IOR/OS-4 [32]. Histologic response to preoperative chemotherapy was evaluated and graded according to previously reported criteria [31].

Surgical excision of the primary tumor involved en bloc resection of the overlying biopsy tract with the tumor. Resection of the tumor and reconstruction of the bone defect were performed according to the operating instructions provided by Enneking et al. [33]. Proximal and distal bony margins were defined by the respective treatment protocol according to Enneking's classification. Detailed bone and soft tissue margin widths were guided by Kawaguchi et al. [34, 35]. The surgical margin was determined by 3 techniques: macroscopic inspection of the specimen, careful dissection, and histologic examination. Tumor size, neurovascular infiltration, and tumor necrosis were also recorded via pathological examination by macroscopic inspection.

Postoperative chemotherapy was given once the wound healed adequately. The chemotherapy protocol was modified if the tumor response was poor according to IOR/OS-4. Postoperative follow-up was performed every 2-3 months in the first two years, every 4 months until 5 years, and every 6 months in the following years. Imaging modalities included bone scans, chest computed tomography (CT) scans, abdominal ultrasounds, and positron emission tomography (PET) scans.

Seventy patients showed good response to neoadjuvant chemotherapy with tumor necrosis >=90%. The average preoperative chemotherapy duration was 10.8 ± 2.1 weeks, and patients were grouped according to their length of preoperative chemotherapy: 110 patients (60.4%) with a duration of 8-12 weeks, 38 patients (20.9%) with a duration greater than 12 weeks, and 34 patients (18.7%) with a duration less than 8 weeks due to noncompliance or severe drug toxicity. Seventy-eight patients (42.9%) underwent primary surgery after 14 days from the end of preoperative chemotherapy, while 77 patients (42.3%) had a delay of greater than twenty-one days to the resumption of chemotherapy (Table 1).

### 2.3. Statistics

Kaplan and Meier estimates were used to assess overall survival (OS) and local recurrence-free overall survival (LRFS) [36]. For survival analysis, the primary end points were time to death, so overall survival was measured from the date of diagnosis to death or the endpoint of follow-up. LRFS was defined as the time from the date of primary surgery to the day when local recurrence was confirmed by biopsy or surgery. Univariate and multivariate Cox proportional hazards regression analyses were performed to determine which parameters were significant. A 95% confidence interval level was used; p < 0.05 was considered as significant to identify factors predictive of local recurrence and overall survival. The multivariate logistic regression model included all the potential predictors. Predictors were excluded (one by one) if the* p* value of the log likelihood ratio test was greater than 0.10. Predictor exclusion was continued until all the remaining predictors had* p* values less than 0.10, which was then defined as the final prediction model. SPSS Version 20.0 (SPSS Inc., Chicago, IL) was used for all statistical analyses.

## 3. Results

### 3.1. Prognostic Factors for Overall Survival

**Figure 1:**
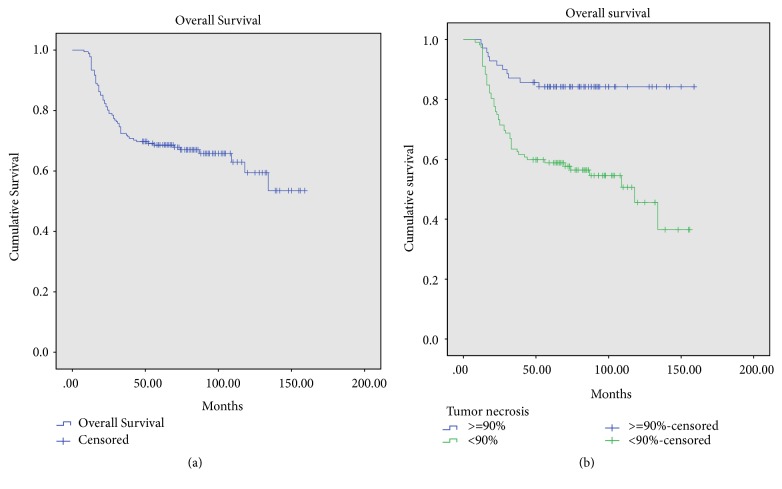
Overall survival for 182 patients and overall survival for patients with tumor necrosis >=90% and not (*p*<0.001).

The 5-year and 10-year overall survival rates were 68.6 ± 6.6% and 59.4 ± 10.6%, respectively (Figure 1). The mean length of follow-up was 73.4 ± 34.7 months (median, 68 months; range, 12-173 months), and 63 patients had died by the end of follow-up.

Univariate analysis showed that the prognostic factors for overall survival were young age at diagnosis, prolonged symptom intervals >=60 days, biopsy/tumor resection performed by different centers, previous medical history, elevated ALP at diagnosis, neurovascular infiltration, tumor size >=10 cm, tumor necrosis <90%, incomplete preoperative chemotherapy (<8 weeks), and prolonged postoperative interval >21 days. In the multivariate analysis, the risk factors were young age at diagnosis, biopsy/tumor resection performed by different centers, elevated ALP at diagnosis, tumor size >=10 cm, tumor necrosis <90%, prolonged postoperative interval >21 days, and shorter preoperative chemotherapy (<8 weeks) (Table 2).

**Figure 2:**
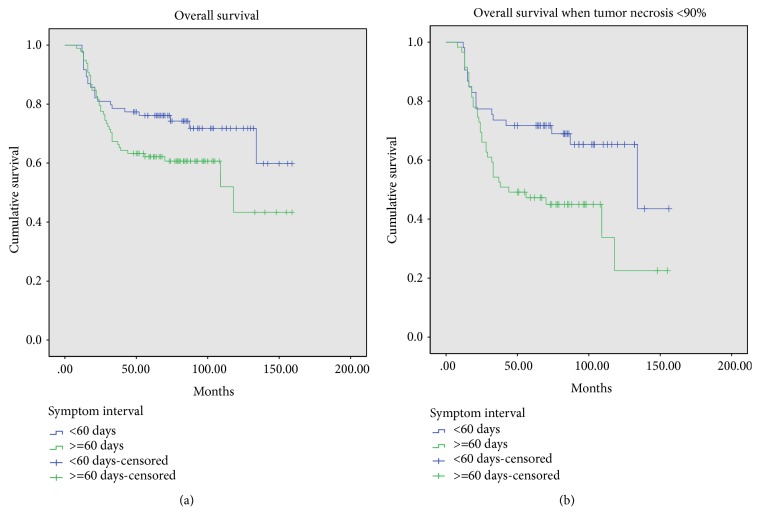
Overall survival for 182 patients with different symptom interval (*p*=0.062) and for 112 patients with tumor necrosis <90% classified with different symptom interval (*p*=0.014).

The 5-year overall survival for patients with tumor necrosis >=90% and patients with tumor necrosis <90% was 84.2±8.6% and 58.9±9.2% (*p*<0.001) (Figure 1). The 5-year overall survival for patients with a prolonged symptom interval (SI>=60 days) and patients with a shorter SI was 62.2±9.6% and 76.1±8%, respectively (*p*=0.062) (Figure 2). Among the 112 patients with tumor necrosis <90%, the 5-year overall survival of 59 patients who had a prolonged symptom interval (SI>=60 days) was 47.4±12.7%, much worse than that (71.7±12.2%) in 53 patients with a shorter SI (*p*=0.014) (log rank test) (Figure 2).

**Figure 3:**
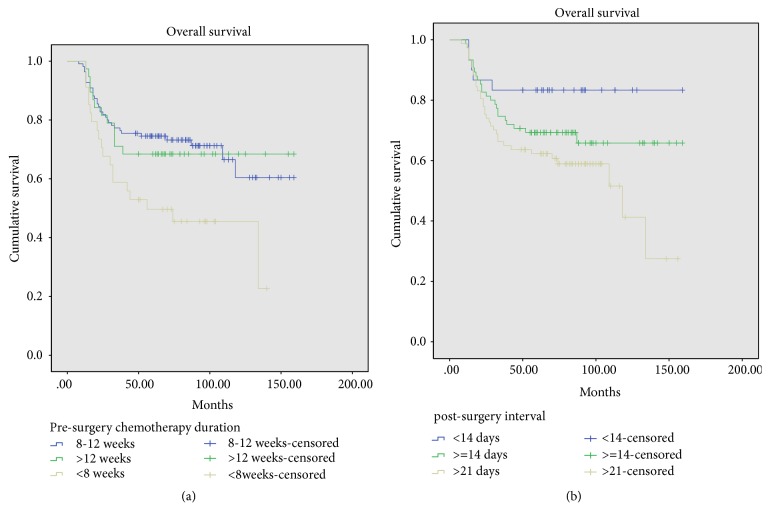
Overall survival for patients with different duration of preoperative chemotherapy (74.5±8.2% (8-12 weeks), 68.4±14.7% (>12 weeks), and 49.6±16.9% (<8 weeks),* p*=0.015) and for patients with different postoperative interval (83.3±13.3% (<14 days), 69.3±10.4% (14-21 days), and 62.3±10.8% (>21 days),* p*=0.041).

The 5-year overall survival rates for patients with different postoperative intervals (PSIs) were 83.3±13.3% (PSI, <14 days), 69.3±10.4% (PSI, 14-21 days), and 62.3±10.8% (*p*=0.041) (Figure 3). The 5-year overall survival rates for patients with different preoperative chemotherapy durations were 74.5±8.2% (8-12 weeks), 68.4±14.7% (>12 weeks), and 49.6±16.9% (<8 weeks) (*p*=0.015) (Figure 3).

### 3.2. Prognostic Factors for Local Recurrence

**Figure 4:**
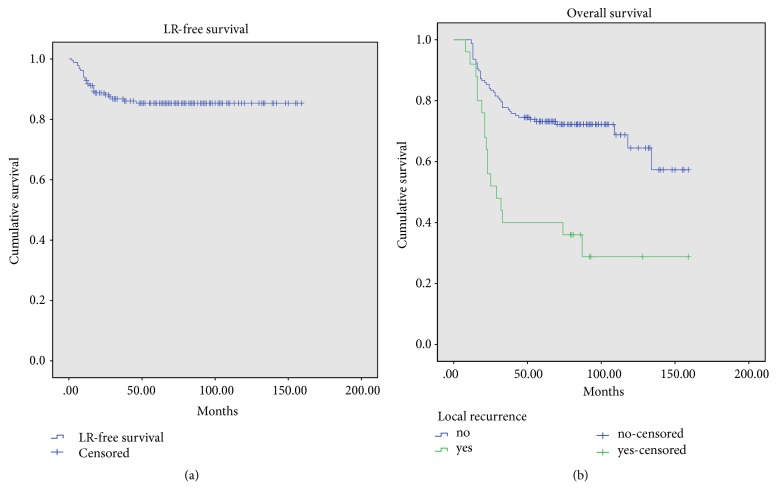
Local recurrence-free survival for 182 patients and overall survival for patients with and without local recurrence (*p*<0.001).

The incidence of local recurrence (LR) in this study was 13.7% (25/182), and the 5-year LR-free survival was 85.3 ± 5.3% (Figure 4). The 5-year overall survival for patients with local recurrence was 40 ± 19.2%, which was significantly lower than that for patients without local recurrence (73.2 ± 6.9%) (*p*<0.001) (Figure 4).

Univariate analysis showed that prognostic factors for LR-free survival were biopsy/tumor resection performed by different centers, neurovascular infiltration, tumor size >=10 cm, and tumor necrosis <90%. Patients with a previous medical history or a larger tumor size were inclined to develop local recurrence, although it was not significant. However, in the multivariate analysis, only biopsy/tumor resection performed by different centers and tumor necrosis <90% were independent predictors of local recurrence. There was no significant difference in the probability regarding sex, age, bone margin width, elevated ALP level at diagnosis, preoperative chemotherapy duration, preoperative interval, and postoperative interval (Table 3).

**Figure 5:**
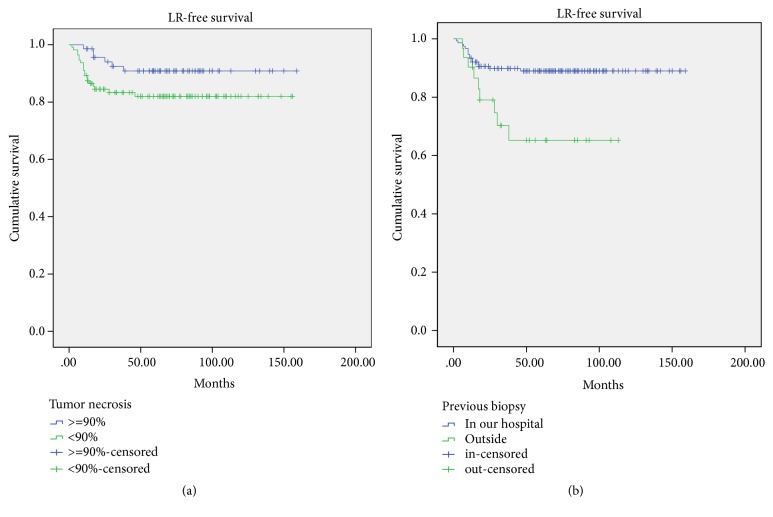
Local recurrence-free survival for patients classified with tumor necrosis (*p*=0.008) and biopsy in same center or not (*p*<0.001).

For patients who underwent biopsy by different centers, their 5-year LR-free survival was 58.1±19.6% and was much worse than that of those whose biopsy was performed in the same center (*p*<0.001) (Figure 5). The 5-year LR-free survival for patients with necrosis <90% was 80.2±7.6%, significantly worse than those with necrosis >=90% (p=0.008) (Figure 5).

## 4. Discussion

In this study, we presented a long-term assessment of overall survival and local recurrence in relation to prognostic factors among patients with nonmetastatic high-grade osteosarcoma around the knee. We found an overall cumulative 5- and 10-year survival rate of 68.6 ± 6.6% and 59.4 ± 10.6%, respectively, with a mean follow-up of 73.4 months. The overall survival in our study is on par with most data [1, 9, 20, 22]. The incidence of local recurrence (LR) in this study was 13.7% (25/182), and the 5-year LR-free survival was 85.3 ± 5.3%. Our local recurrence rate of 13.7% is slightly higher than that presented in some reported data [6, 12, 37, 38], but these data are equal to data from a larger series report [2, 8, 39–41].

In this study, we found that several factors are significant for an unfavorable prognosis after rigorous statistical analysis. These independent factors are listed as follows and coincide with reports from previous literature: young age [9], elevated ALP at diagnosis [9, 42], large tumor size [1, 9, 20, 43], tumor necrosis <90% [1, 8, 12, 18, 43], prolonged postoperative interval >21 days [27], and nonstandard chemotherapy [44, 45]. Sex [8, 22], pathologic fracture [8, 46], and histological subtypes [8, 20, 22] are not prognostic factors, findings which are identical to our outcomes.

Although there is a tendency towards worse overall survival for patients with symptom delays of longer than 60 days, we are unable to find a positive correlation (*p*=0.193, HR=1.443 (0.830-2.509)) in the multivariate analysis between a prolonged SI and overall survival; that is, latency of diagnosis and new adjuvant chemotherapy is not associated with poor prognosis. However, when adjusted for lower tumor necrosis (<90%), patients with an SI longer than 60 days had adverse 5-year overall survival of 47.4±12.7%, which was much worse than that (71.7±12.2%) in patients with a shorter SI (*p*=0.014). This indicates that we should adjust adjuvant therapy and intense surveillance for those patients with a longer SI and a poor chemotherapy response. Similar results that no positive correlation exists between the SI and the outcome of osteosarcoma patients have been shown in other reports [1, 23, 24]. Some studies even show that the SI is shorter in patients with metastatic disease than in patients with localized disease, and patients with a longer duration of symptoms appear to have a better prognosis [47, 48]. Interestingly, Bielack et al. [1] found that longer symptom history and treatment delay do significantly correlate with poor chemotherapy response. Jin et al. [49] concluded that adolescents with longer patient delays may incur inferior prognoses. In this cohort, patients with primary metastases that showed aggressive tumor biology were excluded, but we still found a slight tendency towards a poor prognosis for patients with symptom delays (*p*=0.066, 1.622 (0.969-2.715)) in the univariate analysis. We recommend that we should pay more attention to patients with long symptom intervals. We recommend early diagnosis and treatment to shorten the SI in cases of poor survival because prolonged SI leads to poor chemotherapy response. For patients with prolonged SI at diagnosis, standard chemotherapy should be vigorously executed to obtain good tumor necrosis, avoiding decreasing chemotherapy intensity.

Incomplete preoperative chemotherapy and prolonged preoperative chemotherapy may incur poorer prognosis compared to standard preoperative chemotherapy in our study. Shorter or incomplete preoperative chemotherapy correlates with poor chemotherapy response [50, 51] due to the heterogeneity of patient compliance with chemotherapy or inferior socioeconomic status [44, 45, 52]. Patients with shorter or incomplete preoperative chemotherapy were 2.809 (95% CI, 1.169-6.750) times at risk for poor prognosis in our study. Toxicity, patient choice, and financial inadequacy also resulted in prolonged preoperative chemotherapy duration in our study. Both incomplete preoperative chemotherapy and prolonged preoperative chemotherapy would reduce the overall dose intensity according to recent investigators [53, 54]. The results of dose-intensity analyses performed by other investigators [25, 26, 55, 56] support the hypothesis that the actual dose intensity delivered determines the outcome of treatment of osteosarcoma. On the other hand, some researchers [57, 58] suggest that as preoperative therapy becomes more prolonged, the correlation of tumor necrosis with DFS (disease-free survival) decreases. Standard chemotherapy, if possible, should be rigorous to achieve a superior prognosis.

Similar to the results of Imran et al. [27], the mean surgery to postoperative chemotherapy interval was 23 ± 10.1 days (7-60 days) in our study, and the median length was 21 days. In the study by Imran et al. [27], overall survival was poorer for patients who had a delay of greater than 21 days before the resumption of chemotherapy compared with that of patients who had a shorter delay. Patients with a good response to the preoperative chemotherapy fared worse when the postoperative chemotherapy was delayed for more than sixteen days after the definitive surgery, whereas the impact of delayed resumption of chemotherapy on the patients with a poor response was not significant. The reason may be that a lengthy delay before the resumption of chemotherapy after definitive surgery could compromise the overall dose intensity. Patients with a poor histological response to preoperative chemotherapy have worse survival when they have a delay in resumption of chemotherapy of more than twenty-four days [57]. Our study confirmed these findings in that patients with a postoperative interval >21 days are inclined to incur poor outcomes (HR=2.844 (1.068-7.572),* p*=0.036). In this study, it seems that patients with a postoperative interval longer than 14 days face a negative ending, and the later they resume chemotherapy, the poorer their outcomes are. Whether intensive chemotherapy would impact the chemotherapy response and the survival thereafter is still controversial [53–56]; thus further study on delayed resumption of postoperative chemotherapy and its impact is still needed.

The local recurrence rate in this study is 13.7%, which is slightly higher than the current international standard (4-10%). Neurovascular infiltration [6, 8], large tumor size, and inferior tumor necrosis [38, 59, 60] are commonly regarded as risk factors for local recurrence. Under the promotion of limb-salvage surgery, these factors have been widely and intensely debated [2, 12, 15, 19, 61, 62]. However, in our study, we attribute the higher incidence of local recurrence to inadequate/close margins and poor tumor necrosis. Reasons for inadequate/close margins include unprofessional biopsy and erroneous/unplanned surgery due to an erroneous diagnosis. In this study, 31 patients (17%) received a biopsy in other medical centers and then asked for limb-salvage surgery in our hospital; 17 patients (9%) received prior surgery, such as intralesional/marginal curettage. These unplanned surgeries may lead to problems in managing wide soft tissue margins due to unplanned approaches. That is why all of these recurrent tumors were located in the soft tissue around the wound or near the neurovascular bundle, although frozen-section histology examination by intraoperative biopsies certified that there were no residual tumor cells. A previous medical history due to malignant transformation or erroneous diagnosis and treatment has recently been accepted by some researchers as a risk factor [6, 63–65]. Biopsy/tumor resection performed by different centers in this study is a significant predictor of the development of local recurrence and poorer overall survival. This is similar to the reports by Picci et al. [66], Andreou et al. [6], and Poudel et al. [39]. The biopsy approach must be planned so that it can be completely and widely excised at the moment of the resection. The procedure should be performed by an experienced surgical team who will also be handling the definitive surgery. Poor necrosis due to nonstandard chemotherapy (incomplete chemotherapy) is widely accepted as a risk factor for local recurrence. In this study, 72 patients (39.6%) did not achieve complete or standard chemotherapy due to noncompliance with chemotherapy and low socioeconomic status. Thus, only 70 patients (38.5%) had good tumor necrosis (>=90%). This is one of the reasons for the high incidence of local recurrence in this study.

Due to the lack of uniformity in patient analyses and methods and statistics that were calculated on study populations whose minimum follow-up was often less than 3 years, a number of clinical and pathologic features show bias in different studies and present with contradictory prognostic significance. However, our present analysis evaluated a large number of patients according to the previously mentioned prognostic variables, followed up for at least 5 years. In our study, data about the variables evaluated were available for almost all patients. The main shortcoming is that data were not derived from a randomized study, but we collected information from each patient prospectively. The tumors of the patients included in this study were somehow heterogeneous in terms of biological behavior and stage, but we rigorously set and executed inclusion and exclusion criteria. We believe that our data provide a meaningful contribution to the previous literature.

## 5. Conclusion

For localized osteosarcoma treated with limb-salvage surgery, it is necessary to optimize timely standard chemotherapy and to resume postoperative chemotherapy to improve survival rates. Biopsies should be performed at experienced institutions in cases of developing local recurrence.

## Figures and Tables

**Table 1:** Patient characteristics.

	Number of patients (%)
Gender	
female	68(37.4%)
male	114(62.6%)
Age at diagnosis	21.2±7.6 years, 12-59
31-59 years old	29(15.9%)
21-30 years old	50(27.5%)
10-20 years old	103(56.6%)
Site	
distal femur	118(64.8%)
proximal tibia	64(35.2%)
Symptom intervals	68.2±69.7 days, 23-750
<60 days	84(46.2%)
>=60 days	98(53.8%)
Pathologic fracture	
not occurred	174(95.6%)
occurred	8(4.4%)
Center performing the biopsy/the tumor resection	
same	151(83%)
different	31(17%)
Previous medical history	
no	165(90.7%)
yes	17(9.3%)
Elevated initial ALP	
no	42(23.1%)
yes	140(76.9%)
Bone margin width	
>=3 cm	161(88.5%)
>=2 cm	21(11.5%)
Neurovascular infiltration	
no	122(67%)
yes	60 (33%)
Tumor size	
<10 cm	106(58.2%)
>=10 cm	76(41.8%)
Histological subtypes	
osteoblastic	134(73.6%)
chondroblastic	24(13%)
fibroblastic	10(5.5%)
telengiectatic and other	14(7.9%)
Tumor necrosis	
>=90%	70(38.5%)
<90%	112(61.5%)
Preoperative chemotherapy duration	10.8 ± 2.1weeks, 4-16
8-12 weeks	110(60.4%)
>12 weeks	38(20.9%)
<8 weeks	34(18.7%)
Preoperative interval	13.1 ± 4.1days, 7-25
<14 days	104(57.1%)
>=14 days	78(42.9%)
Postoperative interval	23±10.1 days, 7-60
<=14 days	30(16.5%)
15-21days	75(41.2%)
>21 days	77(42.3%)

**Table 2:** Summary of univariate and multivariate Cox proportional hazards model for overall survival. HR: hazards ratio; LRT: likelihood rate testing.

	Univariate LRT		Multivariate LRT	
	*p* values and HR (95%)		*p* values and HR (95%)	
Gender	0.771			
female	Ref.			
male	0.927(0.557-1.543)			
Age at diagnosis	0.026			
31-59 years old	Ref.			Ref.
21-30 years old	2.849(0.952-8.530)		0.038	2.268(1.065-10.029)
10-20 years old	3.686(1.322-10.283)		0.026	3.304(1.156-9.447)
Site	0.533			
distal femur	Ref.			
proximal tibia	0.845(0.497-1.437)			
Symptom intervals	0.066		0.193	
<60 days	Ref.		Ref.	
>=60 days	1.622(0.969-2.715)		1.443(0.830-2.509)	
Pathologic fracture	0.248			
not occurred	Ref.			
occurred	0.313(0.043-2.260)			
Center performing the biopsy/the tumor resection	0.044		0.001	
same	Ref.		Ref.	
different	1.817(1.017-3.247)		2.797(1.503-5.207)	
Previous medical history	0.045		0.108	
no	Ref.		Ref.	
yes	1.997(1.016-3.927)		1.928(0.865-4.294)	
Elevated ALP at diagnosis	0.041		0.013	
no	Ref.		Ref.	
yes	1.678(1.022-2.756)		2.001(1.156-3.463)	
Bone margin width	0.701			
>=3 cm	Ref.			
>=2 cm	1.157(0.549-2.436)			
Neurovascular infiltration	0.049		0.182	
no	Ref.		Ref.	
yes	1.654(1.003-2.727)		1.479(0.832-2.628)	
Tumor size	0.034		0.003	
<10 cm	Ref.		Ref.	
>=10 cm	1.708(1.041-2.802)		2.199 (1.311-3.690)	
Histological subtypes	0.654			
osteoblastic	Ref.			
chondroblastic	0.765(0.347-1.688)			
fibroblastic	0.458(0.111-1.884)			
telengiectatic and other	0.800(0.289-2.216)			
Tumor necrosis	<0.001		0.001	
>=90%	Ref.		Ref.	
<90%	3.504(1.827-6.720)		3.287(1.665-6.491)	
Preoperative chemotherapy duration	0.019			
8-12 weeks	Ref.		Ref.	
>12 weeks	1.081(0.556-2.099)		0.434	0.745(0.357-1.556)
<8 weeks	2.210(1.251-3.903)		0.008	2.249(1.232-4.105)
Preoperative interval	0.701			
<14 days	Ref.			
>=14 days	0.906(0.547-1.501)			
Postoperative interval	0.051			
<=14 days	Ref.		Ref.	
15-21days	1.965(0.749-5.153)		0.051	2.687(0.998-7.236)
>21 days	2.913(1.139-7.451)		0.036	2.844(1.068-7.572)

**Table 3:** Summary of univariate and multivariate Cox proportional hazards models for LR. HR: hazards ratio; LRT: likelihood rate testing.

	Univariate LRT		Multivariate LRT	
	*p* values and HR (95%)		*p* values and HR (95%)	
Gender	0.241			
female	Ref.			
male	0.625(0.285-1.370)			
Age at diagnosis	0.544			
31-59 years old	Ref.			
21-30 years old	1.947(0.527-7.194)			
10-20 years old	1.366(0.389-4.794)			
Site	0.678			
distal femur	Ref.			
proximal tibia	0.837(0.361-1.940)			
Symptom intervals	0.480			
<60 days	Ref.			
>=60 days	1.334(0.599-2.971)			
Pathologic fracture	0.464			
not occurred	Ref.			
occurred	0.046(0-171.359)			
Center performing the biopsy/the tumor resection	<0.001		0.002	
same	Ref.		Ref.	
different	4.601(2.082-10.169)		4.099(1.649-10.192)	
Previous medical history	0.060		0.931	
no	Ref.		Ref.	
yes	2.561(0.960-6.835)		0.951(0.308-2.942)	
Initial raised ALP	0.301			
no	Ref.			
yes	1.517(0.688-3.344 )			
Bone margin width	0.895			
>=3 cm	Ref.			
>=2 cm	1.084(0.325-3.624)			
Neurovascular infiltration	0.007		0.163	
no	Ref.		Ref.	
yes	4.140(1.826-9.388)		1.838(0.782-4.320)	
Tumor size	0.070		0.152	
<10 cm	Ref.		Ref.	
>=10 cm	2.080(0.043-4.589)		1.871(0.795-4.405)	
Histological subtypes	0.340			
osteoblastic	Ref.			
chondroblastic	0.261(0.035-1.944)			
fibroblastic	1.922(0.571-6.470)			
telengiectatic and other	0.477(0.064-3.555)			
Tumor necrosis	0.014		0.022	
>=90%	Ref.		Ref.	
<90%	3.802(1.304-11.087)		3.536(1.198-10.438)	
Preoperative chemotherapy duration	0.783			
8-12 weeks	Ref.			
>12 weeks	0.694(0.234-2.063)			
<8 weeks	0.818(0.275-2.432)			
Preoperative interval	0.336			
<14 days	Ref.			
>=14 days	1.469(0.670-3.220)			
Postoperative interval	0.761			
<=14 days	Ref.			
15-21days	0.934(0.288-3.034)			
>21 days	1.278(0.412-3.964)			

## Data Availability

The data used to support the findings of this study and the datasets used and/or analyzed during the current study are available from the corresponding author upon request (Chunlin Zhang, shzhangchunlin123@163.com).

## References

[1] Bielack S. S., Kempf-Bielack B., Delling G. (2002). Prognostic factors in high-grade osteosarcoma of the extremities or trunk: an analysis of 1,702 patients treated on neoadjuvant cooperative osteosarcoma study group protocols. *Journal of Clinical Oncology*.

[2] Reddy K. I. A., Wafa H., Gaston C. L. (2015). Does amputation offer any survival benefit over limb salvage in osteosarcoma patients with poor chemonecrosis and close margins?. *The Bone & Joint Journal*.

[3] Han G., Bi W.-Z., Xu M., Jia J.-P., Wang Y. (2016). Amputation versus limb-salvage surgery in patients with osteosarcoma: a meta-analysis. *World Journal of Surgery*.

[4] Mavrogenis A. F., Abati C. N., Romagnoli C., Ruggieri P. (2012). Similar survival but better function for patients after limb salvage versus amputation for distal tibia osteosarcoma. *Clinical Orthopaedics and Related Research*.

[5] Ayerza M. A., Farfalli G. L., Aponte-Tinao L., Luis Muscolo D. (2010). Does increased rate of limb-sparing surgery affect survival in osteosarcoma?. *Clinical Orthopaedics and Related Research*.

[6] Andreou D., Bielack S. S., Carrle D. (2011). The influence of tumor- and treatment-related factors on the development of local recurrence in osteosarcoma after adequate surgery. An analysis of 1355 patients treated on neoadjuvant Cooperative Osteosarcoma Study Group protocols. *Annals of Oncology*.

[7] Li X., Moretti V. M., Ashana A. O., Lackman R. D. (2012). Impact of close surgical margin on local recurrence and survival in osteosarcoma. *International Orthopaedics*.

[8] Puri A., Byregowda S., Gulia A., Crasto S., Chinaswamy G. (2018). A study of 853 high grade osteosarcomas from a single institution—Are outcomes in Indian patients different?. *Journal of Surgical Oncology*.

[9] Bacci G., Longhi A., Versari M., Mercuri M., Briccoli A., Picci P. (2006). Prognostic factors for osteosarcoma of the extremity treated with neoadjuvant chemotherapy: 15-year experience in 789 patients treated at a single institution. *Cancer*.

[10] Bacci G., Mercuri M., Longhi A. (2005). Grade of chemotherapy-induced necrosis as a predictor of local and systemic control in 881 patients with non-metastatic osteosarcoma of the extremities treated with neoadjuvant chemotherapy in a single institution. *European Journal of Cancer*.

[11] Mankin H. J., Hornicek F. J., Rosenberg A. E., Harmon D. C., Gebhardt M. C. (2004). Survival data for 648 patients with osteosarcoma treated at one institution. *Clinical Orthopaedics and Related Research*.

[12] Whelan J. S., Jinks R. C., McTiernan A. (2012). Survival from high-grade localised extremity osteosarcoma: combined results and prognostic factors from three European Osteosarcoma Intergroup randomised controlled trials. *Annals of Oncology*.

[13] Jeys L. M., horne C. J., Parry M., Gaston C. L., Sumathi V. P., Grimer J. R. (2016). A novel system for the surgical staging of primary high-grade osteosarcoma: the birmingham classification. *Clinical Orthopaedics and Related Research*.

[14] Bertrand T. E., Cruz A., Binitie O., Cheong D., Letson G. D. (2016). Do surgical margins affect local recurrence and survival in extremity, nonmetastatic, high-grade osteosarcoma?. *Clinical Orthopaedics and Related Research*.

[15] Loh A. H. P., Wu H., Bahrami A. (2015). Influence of bony resection margins and surgicopathological factors on outcomes in limb-sparing surgery for extremity osteosarcoma. *Pediatric Blood & Cancer*.

[16] Bielack S., Jürgens H., Jundt G. (2009). Osteosarcoma: the COSS experience. *Cancer Treatment and Research*.

[17] Pakos E. E., Nearchou A. D., Grimer R. J. (2009). Prognostic factors and outcomes for osteosarcoma: An international collaboration. *European Journal of Cancer*.

[18] Guadalupe M. P., Boggio G. F., Specterman S., Lastiri J. M., Lincuez M. E. L. (2012). Age as a prognostic factor in osteosarcoma: Survival analysis. *Journal of Clinical Oncology*.

[19] Durnali A., Alkis N., Cangur S. (2013). Prognostic factors for teenage and adult patients with high-grade osteosarcoma: an analysis of 240 patients. *Medical Oncology*.

[20] Duchman K. R., Gao Y., Miller B. J. (2015). Prognostic factors for survival in patients with high-grade osteosarcoma using the Surveillance, Epidemiology, and End Results (SEER) Program database. *Cancer Epidemiology*.

[21] Hung G. Y., Yen H. J., Yen C. C. (2015). Experience of pediatric osteosarcoma of the extremity at a single institution in Taiwan: prognostic factors and impact on survival. *Annals of Surgical Oncology*.

[22] Vasquez L., Tarrillo F., Oscanoa M. (2016). Analysis of prognostic factors in high-grade osteosarcoma of the extremities in children: A 15-year single-institution experience. *Frontiers in Oncology*.

[23] Li H., Zheng S., Yu W. (2016). Symptom interval of osteosarcoma around the knee joint: an analysis of 82 patients of a single institute. *European Journal of Cancer Care*.

[24] Goyal S., Roscoe J., Ryder W. D. J., Gattamaneni H. R., Eden T. O. B. (2004). Symptom interval in young people with bone cancer. *European Journal of Cancer*.

[25] Eselgrim M., Grunert H., Kühne T. (2006). Dose intensity of chemotherapy for osteosarcoma and outcome in the Cooperative Osteosarcoma Study Group (COSS) trials. *Pediatric Blood & Cancer*.

[26] Lewis I., Nooij M. A., Whelan J., Sydes M. R. (2007). Improvement in histologic response but not survival in osteosarcoma patients treated with intensified chemotherapy: a randomized phase III trial of the European Osteosarcoma Intergroup. *Journal of the Egyptian National Cancer Institute*.

[27] Imran H., Enders F., Krailo M. (2009). Effect of time to resumption of chemotherapy after definitive surgery on prognosis for non-metastatic osteosarcoma. *The Journal of Bone and Joint Surgery-American Volume*.

[28] Bajpai J., Puri A., Shah K. (2013). Chemotherapy compliance in patients with osteosarcoma. *Pediatric Blood & Cancer*.

[29] Puri A., Gulia A., Hawaldar R., Ranganathan P., Badwe R. A. (2014). Does intensity of surveillance affect survival after surgery for sarcomas? Results of a randomized noninferiority trial. *Clinical Orthopaedics and Related Research*.

[30] Cipriano C., Griffin A. M., Ferguson P. C., Wunder J. S. (2017). Developing an evidence-based followup schedule for bone sarcomas based on local recurrence and metastatic progression. *Clinical Orthopaedics and Related Research*.

[31] Rosen G., Caparros B., Huvos A. G. (1982). Preoperative chemotherapy for osteogenic sarcoma: selection of postoperative adjuvant chemotherapy based on the response of the primary tumor to preoperative chemotherapy. *Cancer*.

[32] Ferrari S., Smeland S., Mercuri M. (2005). Neoadjuvant chemotherapy with high-dose ifosfamide, high-dose methotrexate, cisplatin, and doxorubicin for patients with localized osteosarcoma of the extremity: a joint study by the italian and Scandinavian Sarcoma Groups. *Journal of Clinical Oncology*.

[33] Enneking W. F., Spanier S. S., Goodman M. A. (1980). A system for the surgical staging of musculoskeletal sarcoma. *Clinical Orthopaedics and Related Research*.

[34] Kawaguchi N., Matumoto S., Manabe J. (1995). New method of evaluating the surgical margin and safety margin for musculoskeletal sarcoma, analysed on the basis of 457 surgical cases. *Journal of Cancer Research and Clinical Oncology*.

[35] Kawaguchi N., Ahmed A. R., Matsumoto S., Manabe J., Matsushita Y. (2004). The concept of curative margin in surgery for bone and soft tissue sarcoma. *Clinical Orthopaedics and Related Research*.

[36] Kaplan E. L., Meier P. (1958). Nonparametric estimation from incomplete observations. *Journal of the American Statistical Association*.

[37] Bacci G., Forni C., Longhi A. (2014). Presentation, treatment, and prognosis of recurrent osteosarcoma: The EUropean RELapsed OsteoSarcoma Registry (EURELOS). *European Journal of Cancer*.

[38] Bacci G., Forni C., Longhi A. (2007). Local recurrence and local control of non-metastatic osteosarcoma of the extremities: a 27-year experience in a single institution. *Journal of Surgical Oncology*.

[39] Poudel R. R., Tiwari V., Kumar V. S. (2017). Factors associated with local recurrence in operated osteosarcomas: A retrospective evaluation of 95 cases from a tertiary care center in a resource challenged environment. *Journal of Surgical Oncology*.

[40] Grimer R. J., Taminiau A. M., Cannon S. R. (2002). Surgical outcomes in osteosarcoma. *The Journal of Bone & Joint Surgery (British Volume)*.

[41] Tsuchiya H., Tomita K. (1992). Prognosis of osteosarcoma treated by limb-salvage surgery: the ten-year intergroup study in Japan. *Japanese Journal of Clinical Oncology*.

[42] Bispo R. Z., de Camargo O. P. (2009). Prognostic factors in the survival of patients diagnosed with primary non- metastatic osteosarcoma with a poor response to neoadjuvant chemotherapy. *Clinics*.

[43] Petrilli A. S., de Camargo B., Filho V. O., Bruniera P. (2006). Results of the brazilian osteosarcoma treatment group studies iii and iv: prognostic factors and impact on survival. *Journal of Clinical Oncology*.

[44] Faisham W. I., Mat Saad A. Z., Alsaigh L. N. (2017). Prognostic factors and survival rate of osteosarcoma: A single-institution study. *Asia-Pacific Journal of Clinical Oncology*.

[45] Pruksakorn D., Phanphaisarn A., Arpornchayanon O., Uttamo N., Leerapun T., Settakorn J. (2015). Survival rate and prognostic factors of conventional osteosarcoma in Northern Thailand: A series from Chiang Mai University Hospital. *Cancer Epidemiology*.

[46] Xie L., Guo W., Li Y., Ji T., Sun X. (2012). Pathologic fracture does not influence local recurrence and survival in high-grade extremity osteosarcoma with adequate surgical margins. *Journal of Surgical Oncology*.

[47] Nataraj V., Batra A., Rastogi S. (2015). Developing a prognostic model for patients with localized osteosarcoma treated with uniform chemotherapy protocol without high dose methotrexate: A single-center experience of 237 patients. *Journal of Surgical Oncology*.

[48] Grimer R. J. (2006). Size matters for sarcomas!. *Annals of the Royal College of Surgeons of England*.

[49] Jin S. L., Hahn S. M., Kim H. S. (2016). Symptom interval and patient delay affect survival outcomes in adolescent cancer patients. *Yonsei Medical Journal*.

[50] Miller B. J., Gao Y., Duchman K. R. (2017). Socioeconomic measures influence survival in osteosarcoma: an analysis of the National Cancer Data Base. *Cancer Epidemiology*.

[51] Moreno F., Cacciavillano W., Cipolla M. (2017). Childhood osteosarcoma: Incidence and survival in Argentina. Report from the National Pediatric Cancer Registry, ROHA Network 2000-2013. *Pediatric Blood & Cancer*.

[52] Tan P. X., Yong B. C., Wang J. (2012). Analysis of the efficacy and prognosis of limb-salvage surgery for osteosarcoma around the knee. *European Journal of Surgical Oncology*.

[53] Meyers P. A., Gorlick R., Heller G. (1998). Intensification of preoperative chemotherapy for osteogenic sarcoma: Results of the Memorial Sloan-Kettering (T12) protocol. *Journal of Clinical Oncology*.

[54] Bishop M. W., Chang Y.-C., Krailo M. D. (2016). Assessing the prognostic significance of histologic response in osteosarcoma: a comparison of outcomes on CCG-782 and INT0133—a report from the children's oncology group bone tumor committee. *Pediatric Blood & Cancer*.

[55] Bacci G., Picci P., Avella M. (1990). The importance of dose-intensity in neoadjuvant chemotherapy of osteosarcoma: A retrospective analysis of high-dose methotrexate, cisplatinum and adriamycin used preoperatively. *Journal of Chemotherapy*.

[56] Bacci G., Ferrari S., Longhi A., Forni C. (2001). Relationship between dose-intensity of treatment and outcome for patients with osteosarcoma of the extremity treated with neoadjuvant chemotherapy. *Oncology Reports*.

[57] Meyers P. A., Heller G., Healey J. (1992). Chemotherapy for nonmetastatic osteogenic sarcoma: the memorial sloan-kettering experience. *Journal of Clinical Oncology*.

[58] Simone J. V., Meyer W. H., Link M. P. (1992). Osteosarcoma: Good news despite crude tools. *Journal of Clinical Oncology*.

[59] Poudel R. R., Kumar V. S., Bakhshi S., Gamanagatti S., Rastogi S., Khan S. A. (2014). High tumor volume and local recurrence following surgery in osteosarcoma: A retrospective study. *Indian Journal of Orthopaedics*.

[60] Song W. S., Jeon D.-G., Kong C.-B. (2011). Tumor volume increase during preoperative chemotherapy as a novel predictor of local recurrence in extremity osteosarcoma. *Annals of Surgical Oncology*.

[61] Xu M., Xu S., Yu X. (2014). Marginal resection for osteosarcoma with effective neoadjuvant chemotherapy: Long-term outcomes. *World Journal of Surgical Oncology*.

[62] Jeon D.-G., Song W. S., Kong C.-B. (2013). Role of surgical margin on local recurrence in high risk extremity osteosarcoma: a case-controlled study. *Clinics in Orthopedic Surgery*.

[63] Gaston C. L., Nakamura T., Reddy K. (2014). Is limb salvage surgery safe for bone sarcomas identified after a previous surgical procedure?. *The Bone & Joint Journal*.

[64] Ayerza M. A., Muscolo D. L., Aponte-Tinao L. A., Farfalli G. (2006). Effect of erroneous surgical procedures on recurrence and survival rates for patients with osteosarcoma. *Clinical Orthopaedics and Related Research*.

[65] Wang B., Xu M., Zheng K., Yu X. (2016). Effect of Unplanned Therapy on the Prognosis of Patients with Extremity Osteosarcoma. *Scientific Reports*.

[66] Picci P., Sangiorgi L., Bahamonde L. (1997). Risk factors for local recurrences after limb-salvage surgery for high-grade osteosarcoma of the extremities. *Annals of Oncology*.

